# Queues with Dropping Functions and General Arrival Processes

**DOI:** 10.1371/journal.pone.0150702

**Published:** 2016-03-04

**Authors:** Andrzej Chydzinski, Pawel Mrozowski

**Affiliations:** Institute of Informatics, Silesian University of Technology, Gliwice, Poland; Tianjin University of Science and Technology, CHINA

## Abstract

In a queueing system with the dropping function the arriving customer can be denied service (dropped) with the probability that is a function of the queue length at the time of arrival of this customer. The potential applicability of such mechanism is very wide due to the fact that by choosing the shape of this function one can easily manipulate several performance characteristics of the queueing system. In this paper we carry out analysis of the queueing system with the dropping function and a very general model of arrival process—the model which includes batch arrivals and the interarrival time autocorrelation, and allows for fitting the actual shape of the interarrival time distribution and its moments. For such a system we obtain formulas for the distribution of the queue length and the overall customer loss ratio. The analytical results are accompanied with numerical examples computed for several dropping functions.

## 1 Introduction

Consider a simple queueing system with a stream of arriving customers, the queue of customers waiting for service and one service station (server) performing the service that takes random time. In order to control the performance of such system (e.g. to keep the mean queue length below 10) we have three options:

(a) manipulate dynamically the service rate;
(b) manipulate dynamically the customer arrival rate;
(c) deny the service to some customers and not allow them to the queue.

All the listed approaches are equally good for control purposes in the sense that we can achieve the same results using each of them. The difference between them is in the practical applicability, which depends on the character and purpose of the queueing system of interest.

In many everyday-life queues, approach (a) can be used. At a bank for instance, additional cashiers can be assigned for serving an exceptionally long queue, thus multiplying the service rate. For serving exceptional passenger traffic, a bus (train, ferry) of larger capacity can be used occasionally, etc.

Usually, the application of (a) is connected with some additional costs (salary, equipment, energy consumption etc.). Of course, it can be applied only from time to time, using some sophisticated policy based on current queue size or predictions on its future size.

In some cases however, it is not possible to use (a). In networking for instance, when a queue of packets is being transmitted at a router’s output interface, it is not possible to enlarge the throughput of the physical output link on demand.

Approach (b) cannot be used in most everyday-life applications of queueing systems. This is due to the fact, that the operator of the queue has usually no means to reduce quickly the customer arrival rate (think of a bank, for instance). On the other hand, method (b) works very well in networking. In particular, the Internet hosts, which use the TCP protocol, are forced to reduce their packet sending rates immediately, when the network congestion is expected. This proved to be an efficient way for preventing congestion collapses of the Internet, which were observed in its early years of operation.

As for (c), it is certainly the simplest method. Rejecting a customer is not technically difficult and does not require any spare resources, as method (a). Its disadvantage is that a fraction of customers leaves the system unserved. Therefore, it can be used in these applications only, in which such losses are tolerated. This is the case of the Internet, where packet losses are unavoidable anyway due to buffer overflows and traffic burstiness, so they may be as well caused rather in a planned, controlled manner. There are also many everyday-life examples, in which approach (c) can be used. For instance, when a waiting line at a call center is long, it might be better to reject a customer at once, instead of allowing him to the queue and keeping online for a long time before serving. Of course, there are also every-day life examples in which customers cannot be rejected—in those cases we are practically limited to method (a).

The queueing models of type (a) and their solutions can be found, for instance, in [1–4], while the models of type (b), in [5–7].

In this paper we deal with type (c) of controlling the performance of a queueing system. The literature on this method will be discussed later.

**Fig 1:**
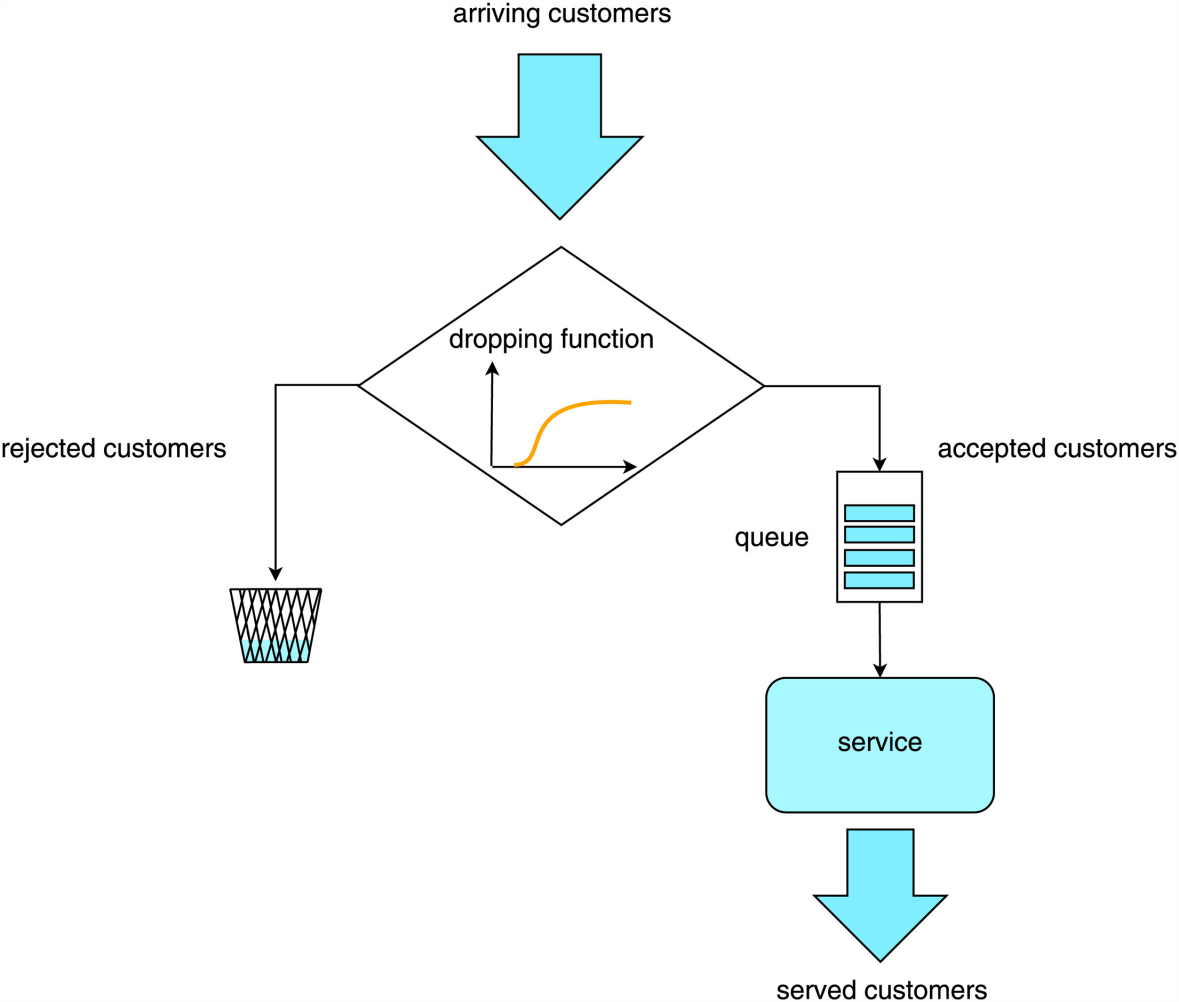
The idea of the queueing system with the dropping function.

In order to decide in method (c), whether a customer has to be accepted to the queue or rejected, a large number of different disciplines can be proposed. One of the most natural ones is that each customer can be rejected randomly, with the probability that depends on the length of the queue upon the arrival of this customer (see Fig 1). In this paper we deal with such discipline. The function mapping the queue lengths into probabilities is called the dropping function and will be denoted by *d*(*n*), where *n* is the queue length. In most practical situations the dropping function would be non-decreasing, i.e. the longer the queue, the more likely the customer is rejected. (However, this assumption is not necessary in our analysis).

The idea of a queueing system with the dropping function was for the first time proposed in networking, in the RED algorithm [8], which uses linear dropping function to drop packets incoming to an Internet router. Following the first, linear dropping function, some other types of functions were studied: doubly linear [9], exponential [10] and polynomial [11]. In addition, a large number of studies were carried out on systems, in which the packet dropping probability is not a simple function of the queue length, but depends on several other factors, often in a very complex way (see e.g. [12–20]). In networking, this is called the active queue management (AQM).

However, besides networking, the dropping functions have great potential for applications in many other systems involving queueing of customers (or jobs, tasks etc.). This is connected with their powerful control capabilities. Namely, by shaping the dropping function according to our needs, we can keep the mean queue length, its variance, the loss ratio and several other performance characteristics at some required levels.

The literature on analytical solutions of queueing systems with dropping functions is not extensive. What is important, all the previous works were devoted to systems with very simple arrival processes, namely Poisson or renewal processes without interarrival times correlation. In particular, in [21], an approximate analysis of the queue with batch Poisson arrivals, linear dropping function and exponential service times was carried out. In [22], an approximate analysis of the system with batch renewal arrivals and exponential service times was performed. In [23] an exact analysis of the queue with Poisson arrivals, arbitrary dropping function and arbitrary service times was presented for systems in the equilibrium. In [24], an exact analysis of the queue with renewal arrivals, arbitrary dropping function and exponential service times was shown. Then, in [25], an analysis of the system with Poisson arrivals and arbitrary dropping function was carried out in the transient case. Finally, in [26] a solution of the system with Poisson arrivals and general distribution of the job size was shown.

Due to the simplicity of the arrival processes, all the mentioned models are not adequate for modeling of many real systems. For instance, it is well known that streams of packets in the Internet have strongly autocorrelated structure (see e.g. [27–29])—not only are the interarrival times correlated, but also the packet sizes may be correlated with the local intensity of the arrival stream. On the other hand, it is well known that not taking these properties into account may lead to an optimistic underestimation of the queueing characteristics by several orders of magnitude (see e.g. [30]). For these reasons, the previous analytical results on queues with dropping functions are of little use in networking and in other applications with sophisticated, autocorrelated traffic of customers.

The novelty of this paper is that we carry out analysis of the system with:

- a very general model of the arrival process, taking into account the correlation of the interarrival times, correlation between local rate and job (customer) size, batch arrivals, the actual shape of the interarrival time distribution and its moments;
- arbitrary distribution of the customer service time;
- arbitrary dropping function.

To the best of the authors’ knowledge, there are no results of this generality in the literature.

Given the presented requirements, a rather obvious choice of the model of the arrival process is the batch Markovian arrival process (BMAP, see [31]). It not only fulfills all the presented requirements, but also is analytically tractable and several efficient procedures for fitting precisely the BMAP parameters to the real, observed arrival processes have been proposed, [32–35].

As for the queueing characteristics of interest, we present analytical formulas for the two most important ones: the queue length distribution and the loss ratio (the fraction of rejected customers). The results on the queue length are presented in the form which allows obtaining both transient and stationary distributions.

In addition to analytical solutions, we present numerical examples for several dropping functions.

The remaining part of the paper is structured as follows. In Section 2, a formal description of the queueing system is presented, including the definition and basic characteristics of the batch Markovian arrival process. Then, in Section 3, the main results on the distribution of the queue length and loss ratio are proven. In Section 4, calculations of two auxiliary functions, *H* and *q*, needed to obtain numerical values of queue lengths and loss ratios, are presented. In Section 5, numerical examples are shown. Finally, remarks concluding the paper are gathered in Section 6.

## 2 Model of the queue

We deal with a queueing system with single service station, whose arrival process is the batch Markovian arrival process (defined below). The customers are served in the arrival order; those who cannot be served immediately form a queue. The service time is random and can have an arbitrary distribution, with distribution function denoted by *F*(*t*). It is assumed, that the capacity of the waiting room (buffer) is limited and equal to *b* customers. This means that the number of customers present in the system, in the queue and the service position, must not exceed *b*. A customer who arrives when the waiting room is full is rejected and never returns.

Moreover, a customer who arrives when the waiting room is not full can be rejected with probability *d*(*n*), where *n* is the queue length at the time of arrival of this customer (including the service position, if occupied). Function *d*(*n*) will be called the dropping function. The dropping function assumes values in [0, 1] for *n* = 0, …, *b* − 1. As the waiting room is finite, the dropping function must fulfill the condition: *d*(*n*) = 1 for *n* ≥ *b*. The queue length (including the service position, if occupied) at time *t* will be denoted by *X*(*t*). If *X*(0) > 0, then it is assumed that the time origin corresponds to a service completion. The load offered to the queueing system is defined in a natural way as:

$$\begin{array}{c} \rho =\Lambda \int_{0}^{\infty }xdF\left(x\right), \end{array}$$

where Λ is the average rate of the arrival process, given by Eq (5).

The batch Markovian arrival process was proposed (using different parameterization) in [36] and initially called N-process. An easier-to-use parameterization of it was introduced in [31]—since then the acronym BMAP has been used. A rich description of the BMAP process, its characteristics and the bibliography can be found in [37].

Let *I* be the unit matrix of size *m* × *m*, **0** be the square matrix of zeroes, while **1** be the column vector of size *m* with all entries equal to 1.

BMAP is defined as the two-dimensional Markov chain (*N*(*t*), *J*(*t*)) on state space {(*i*, *j*):*i* ≥ 0, 1 ≤ *j* ≤ *m*}, with the intensity matrix *Q* in the following form:

$$\begin{array}{c} Q=\left[\begin{array}{ccccc} D_{0} & D_{1} & D_{2} & D_{3} & \cdot \;\cdot \\ & D_{0} & D_{1} & D_{2} & \cdot \;\cdot \\ & & D_{0} & D_{1} & \cdot \;\cdot \\ & & & \cdot & \cdot \;\cdot \end{array}\right], \end{array}$$

where *D*_*k*_, *k* ≥ 0, are *m* × *m* matrices. Moreover, *D*_*k*_ for *k* ≥ 1 are non-negative, *D*_0_ has negative entries on its diagonal and non-negative elsewhere, matrix *D* defined as

$$\begin{array}{c} D=\sum_{k=0}^{\infty }D_{k} \end{array}$$

is an irreducible intensity matrix and *D* ≠ *D*_0_.

The components of the Markov chain (*N*(*t*), *J*(*t*)) are to be interpreted as follows: *N*(*t*) is the total number of customers arriving in interval (0, *t*], while *J*(*t*) is the state at time *t* of the one-dimensional Markov chain modulating the customer arrival process, whose intensity matrix is *D*. The stationary distribution of *J*(*t*) will be denoted by *π*, and, as always, we have:

$$\begin{array}{c} \pi D=[0,\ldots ,0],\;\;\;\pi 1=1. \end{array}$$

The evolution of the BMAP process can be presented in the following manner. Say, at *t* = 0 the modulating chain *J* is in some state *i*. The modulating chain remains in this state for some random time, which is exponentially distributed with mean 1/*μ*_*i*_ where

$$\begin{array}{c} \mu_{i}=-{\left(D_{0}\right)}_{ii}. \end{array}$$ (1)

After this random time, the state of the modulating chain changes into *k*, possibly with an arrival of a batch of *j* customers. Precisely, this happens with probability *p*_*i*_(*j*, *k*), where:

$$\begin{array}{c} p_{i}\left(0,i\right)=0,\;\;\;\;\;\;1\leq i\leq m, \end{array}$$ (2)

$$\begin{array}{c} p_{i}\left(0,k\right)=\frac{1}{\mu_{i}}{\left(D_{0}\right)}_{ik},\;\;\;\;\;\;\;1\leq i,k\leq m,\;\;\;k\neq i, \end{array}$$ (3)

$$\begin{array}{c} p_{i}\left(j,k\right)=\frac{1}{\mu_{i}}{\left(D_{j}\right)}_{ik},\;\;\;\;\;\;\;1\leq i,k\leq m,\;\;\;j\geq 1. \end{array}$$ (4)

(As it is possible that *p*_*i*_(0, *k*) > 0, a change of the modulating state without arrival of any customers can happen as well). After the change, the modulating chain remains in state *k* for some random time, which is exponentially distributed with mean 1/*μ*_*k*_, and so on.

The most important characteristics of the BMAP process can be computed as follows. The average rate of the process, Λ, is

$$\begin{array}{c} \Lambda =\pi \sum_{k=1}^{\infty }kD_{k}1, \end{array}$$ (5)

while the average rate of arrivals of batches is

$$\begin{array}{c} \Lambda_{g}=\pi \left(-D_{0}\right)1. \end{array}$$

Therefore, the average size of a batch is equal to

$$\begin{array}{c} \eta =\frac{\Lambda }{\Lambda_{g}}. \end{array}$$

The variance of the time between arrivals of consecutive batches equals

$$\begin{array}{c} Var=-\frac{2}{\Lambda_{g}}\pi D_{0}^{-1}1-\frac{1}{\Lambda_{g}^{2}}. \end{array}$$

The *k*-lag autocorrelation of batch interarrival times is

$$\begin{array}{c} Corr\left(k\right)=p\;D_{0}^{-1}C\left(C^{k-1}-1\;p\right)D_{0}^{-1}C\;1/Var, \end{array}$$

with $C=-D_{0}^{-1}\left(D-D_{0}\right),$ where *p* is the stationary vector for intensity matrix *C*−*I*, i.e. it fulfills

$$\begin{array}{c} p(C-I)=[0,\ldots ,0],\;\;\;p\;1=1. \end{array}$$

The counting function of the BMAP process is defined as

$$\begin{array}{c} P_{i,j}\left(n,t\right)=P\left(N\left(t\right)=n,J\left(t\right)=j|N\left(0\right)=0,J\left(0\right)=i\right), \end{array}$$

where P denotes probability. Using the matrix notation, we can denote the counting functions as

$$\begin{array}{c} P\left(n,t\right)={\left[P_{i,j}\left(n,t\right)\right]}_{i,j},\;\;\;1\leq i,j\leq m. \end{array}$$

The generating function of the counting function is then

$$\begin{array}{c} P^{*}\left(z,t\right)=\sum_{n=0}^{\infty }P\left(n,t\right)z^{n}=e^{D(z)t}, \end{array}$$

where

$$\begin{array}{c} D\left(z\right)=\sum_{k=0}^{\infty }z^{k}D_{k},\;\;\;\left|z\right|\leq 1. \end{array}$$

BMAP processes have been successfully used in the modeling of traffic in telecommunication networks, e.g. [38–40], as well as of vehicular traffic, [41].

As regards previous research on queueing models with BMAP arrivals, but without the dropping function, in [31, 42] the steady-state analysis of models with infinite buffers was carried out. In [43–45], the transient states of queues with infinite buffers were studied. The systems with the finite waiting room were studied in steady state in [46–48], while in transient case in [49, 50], using the potential method (see also [51]).

## 3 Queue length distribution and losses

We will denote by Φ_*n*, *i*_(*t*, *l*) the probability that the queue length at time *t* is *l*, under assumption that at the beginning (*t* = 0) the queue length was *n*, and the state of the modulating chain was *i*:

$$\begin{array}{c} \Phi_{n,i}\left(t,l\right)=P\left(X\left(t\right)=l|X\left(0\right)=n,J\left(0\right)=i\right), \end{array}$$

where 0 ≤ *n* ≤ *b*, 1 ≤ *i* ≤ *m*, *t* > 0, 0 ≤ *l* ≤ *b*.

A very import role in the computation of Φ_*n*, *i*_(*t*, *l*) will be played by two functions: *H*_*n*,*k*,*i*,*j*_(*u*) and *q*_*n*_(*v*,*k*).

Firstly, *H*_*n*,*k*,*i*,*j*_(*u*) is the counting function of the arrival process filtered by the dropping mechanism. It is defined as the probability that in a system with suspended service, exactly *k* customers would be accepted into the waiting room in time interval (0, *u*] and there would be *J*(*u*) = *j*, assuming that initially there was *X*(0) = *n* and *J*(0) = *i*. In this definition, the suspended service means that the number of customers present in the queue follows from the BMAP arrivals and the dropping mechanism only; the service is turned off and no served customers leave the system in interval (0, *u*].

Secondly, *q*_*n*_(*v*, *k*) denotes the probability, that when a batch of *v* customers arrives to the system in which *n* customers are already present, exactly *k* customers from this batch are accepted. The fact that sometimes only a part of the batch is accepted is, of course, a consequence of the assumed dropping policy and finite waiting room.

Both *H*_*n*,*k*,*i*,*j*_(*u*) and *q*_*n*_(*v*, *k*) will be computed in Section 4.

Having defined *H*_*n*,*k*,*i*,*j*_(*u*) and *q*_*n*_(*v*, *k*), we can build a system of integral equations for function Φ_*n*, *i*_(*t*, *l*). If at *t* = 0 there is at least one customer in the system, the total probability formula used with respect to the first arrival time, *u*, allows us to write:

$$\begin{array}{c} \Phi_{n,i}\left(t,l\right)=\sum_{j=1}^{m}\sum_{k=0}^{b-n}\int_{0}^{t}H_{n,k,i,j}\left(u\right)\Phi_{n+k-1,j}\left(t-u,l\right)dF\left(u\right)+\rho_{n,i}\left(t,l\right), \end{array}$$ (6)

where 1 ≤ *n* ≤ *b*, 1 ≤ *i* ≤ *m* and

$$\begin{array}{c} \rho_{n,i}\left(t,l\right)=\left\{\begin{array}{ccc} 0, & \;\text{if} & \;\;\;\;l<n,\\ \left(1-F\left(t\right)\right)\sum_{j=1}^{m}H_{n,l-n,i,j}\left(t\right), & \;\text{if} & \;\;\;\;n\leq l\leq b. \end{array}\right. \end{array}$$

In the first sum on the right-hand side of Eq (6), all the events in which the first service completion time, *u*, happens before *t*, are taken into account. On the other hand, in function *ρ*_*n*, *i*_(*t*, *l*) all the events in which there is no service completion by the time *t* are taken into account.

If at *t* = 0 there are no customers in the system, the total probability formula used with respect to the first event in the arrival process, gives:

$$\begin{array}{l} \begin{array}{ll} \Phi_{0,i}\left(t,l\right)= & \overset{m}{\underset{j=1}{\sum }}\overset{b}{\underset{k=0}{\sum }}\overset{\infty }{\underset{v=0}{\sum }}\overset{t}{\underset{0}{\int }}\Phi_{k,j}\left(t-u,l\right)q_{0}\left(v,k\right)p_{i}\left(v,j\right)\mu_{i}e^{-\mu_{i}u}du\\ & +\delta_{0l}e^{-\mu_{i}t}, \end{array} \end{array}$$ (7)

where 1 ≤ *i* ≤ *m* and *δ*_*ij*_ equals 1 if *i* = *j* and 0 otherwise. As presented in the definition of BMAP, the first event in BMAP happens after an exponentially distributed time with density *μ*_*i*_ *e*^−*μ*_*i*_*u*^. The first summand of Eq (7) corresponds to the case *u* < *t*. The first event in BMAP can be an arrival of a batch of size *v* or a change of the modulating state into *j*, or both. If a batch of *v* customers arrives indeed, *k* of these customers are accepted with probability *q*_0_(*v*, *k*). Therefore, at time *u* we have *k* customers in the system and the state of the modulating chain is *j*. The second summand of Eq (7) corresponds to the case, where the first event in BMAP is after *t*. In this case the queue length at time *t* is equal to 0.

Applying the following notation

$$\begin{array}{c} a_{n,k,i,j}\left(s\right)=\int_{0}^{\infty }e^{-st}H_{n,k,i,j}\left(t\right)dF\left(t\right), \end{array}$$

$$\begin{array}{c} d_{n,k,i,j}\left(s\right)=\int_{0}^{\infty }e^{-st}H_{n,k,i,j}\left(t\right)\left(1-F\left(t\right)\right)dt, \end{array}$$

and using the Laplace transform to Eqs (6) and (7) yields:

$$\begin{array}{c} \phi_{n,i}\left(s,l\right)=\sum_{j=1}^{m}\sum_{k=0}^{b-n}a_{n,k,i,j}\left(s\right)\phi_{n+k-1,j}\left(s,l\right)+\int_{0}^{\infty }e^{-st}\rho_{n,i}\left(t,l\right)dt,\;\;1\leq n\leq b,1\leq i\leq m, \end{array}$$ (8)

$$\begin{array}{c} \phi_{0,i}\left(s,l\right)=\sum_{j=1}^{m}\sum_{k=0}^{b}\sum_{v=0}^{\infty }\phi_{k,j}\left(s,l\right)q_{0}\left(v,k\right)p_{i}\left(v,j\right)\frac{\mu_{i}}{s+\mu_{i}}+\delta_{0l}\frac{1}{s+\mu_{i}},\;\;1\leq i\leq m, \end{array}$$ (9)

respectively. Now we will rewrite Eqs (8) and (9) in the matrix form. The following square matrices will be used for this purpose:

$$\begin{array}{c} A_{n,k}\left(s\right)={\left[a_{n,k,i,j}\left(s\right)\right]}_{i,j},\;\;\;\;\;1\leq i,j\leq m, \end{array}$$ (10)

$$\begin{array}{c} {\bar{D}}_{n,k}\left(s\right)={\left[d_{n,k,i,j}\left(s\right)\right]}_{i,j},\;\;\;\;\;1\leq i,j\leq m, \end{array}$$ (11)

$$\begin{array}{c} K_{n,k}\left(s\right)=\sum_{v=0}^{\infty }{\left[\frac{q_{n}\left(v,k\right)p_{i}\left(v,j\right)\mu_{i}}{s+\mu_{i}}\right]}_{i,j},\;\;\;\;\;1\leq i,j\leq m, \end{array}$$ (12)

$$\begin{array}{c} 0={\left[0\right]}_{i,j},\;\;\;\;\;1\leq i,j\leq m, \end{array}$$ (13)

as well as column vectors:

$$\begin{array}{c} \phi_{n}\left(s,l\right)={\left[\phi_{n,1}\left(s,l\right),\ldots ,\phi_{n,m}\left(s,l\right)\right]}^{T},\;\;\;\phi_{n,i}\left(s,l\right)=\int_{0}^{\infty }e^{-st}\Phi_{n,i}\left(t,l\right)dt, \end{array}$$ (14)

$$\begin{array}{c} z\left(s\right)={\left[\frac{1}{s+\mu_{1}},\;\ldots \;,\frac{1}{s+\mu_{m}}\right]}^{T}, \end{array}$$ (15)

$$\begin{array}{c} r_{n}\left(s,l\right)=\left\{\begin{array}{ccc} 0\cdot 1, & \;\;\text{if}\; & \;l<n,\\ {\bar{D}}_{n,l-n}\left(s\right)\cdot 1, & \;\;\text{if}\; & \;n\leq l\leq b. \end{array}\right. \end{array}$$ (16)

Using the introduced notation we can rewrite Eqs (8) and (9) as

$$\begin{array}{c} \phi_{n}\left(s,l\right)=\sum_{k=0}^{b-n}A_{n,k}\left(s\right)\phi_{n+k-1}\left(s,l\right)+r_{n}\left(s,l\right),\;\;\;\;\;\;1\leq n\leq b, \end{array}$$ (17)

$$\begin{array}{c} \phi_{0}\left(s,l\right)=\sum_{k=0}^{b}K_{0,k}\left(s\right)\phi_{k}\left(s,l\right)+\delta_{0l}z\left(s\right), \end{array}$$ (18)

respectively.

Introducing the following (*b* + 1)*m* × (*b* + 1)*m* matrix:

$$\begin{array}{c} M\left(s\right)={\left[M_{ij}\left(s\right)\right]}_{i=0\ldots b,j=0\ldots ,b}, \end{array}$$

where

$$\begin{array}{c} M_{ij}\left(s\right)=\left\{\begin{array}{ccc} K_{0,0}\left(s\right)-I, & \;\text{if} & i=0,j=0,\\ K_{0,j}\left(s\right), & \;\text{if} & i=0,1\leq j\leq b,\\ A_{i,j-i+1}\left(s\right), & \;\text{if} & 1\leq i\leq b-2,i<j\leq b-1,\\ A_{i,1}\left(s\right)-I, & \;\text{if} & i=j,1\leq i\leq b-1,\\ A_{i,0}\left(s\right), & \;\text{if} & i=j+1,\\ -I, & \;\text{if} & i=b,j=b,\\ 0, & \text{otherwise}, & \end{array}\right. \end{array}$$ (19)

system Eqs (17) and (18) becomes equivalent to

M(s)ϕ(s,l)=R(s,l), (20)

where *R*(*s*, *l*), *ϕ*(*s*, *l*) are column vectors of size (*b* + 1)*m* defined as follows:

$$\phi (s,l)={\left[\phi_{0}(s,l),\ldots ,\phi_{b}(s,l)\right]}^{T},$$ (21)

$$R(s,l)={\left[R_{0}(s,l),\ldots ,R_{b}(s,l)\right]}^{T},\;\;R_{i}(s,l)=\{\begin{array}{lll} -\delta_{0l}z(s), & \text{ if} & \;\;i=0,\\ -r_{i}(s,l), & \text{ if} & \;\;1\leq i\leq b. \end{array}$$ (22)

Finally, rewriting Eq (20), the following theorem has been proven.

**Theorem 1.** *The Laplace transform of the queue length distribution at time t of a finite-buffer queue with BMAP arrivals and the dropping function equals*:

$$\begin{array}{c} \phi \left(s,l\right)=M^{-1}\left(s\right)R\left(s,l\right),\;\;\;\;0\leq l\leq b, \end{array}$$ (23)

*where M*(*s*) *and R*(*s*, *l*) *are given in formulas* (19) *and* (22), *respectively*.

Using Theorem 1 we can obtain both the transient and stationary distribution of the queue length. For calculations of the transient distribution, we have to exploit one of the several available methods for numerical inversion of the Laplace transform (see e.g [52–56]). The stationary distribution does not need that. It can be obtained directly from Eq (23) by using the basic properties of the Laplace transform. Namely, we have

$$\begin{array}{c} P_{l}=\lim_{t\to \infty }P\left(X\left(t\right)=l\right)=\lim_{s\to 0+}s{\left[M^{-1}\left(s\right)R\left(s,l\right)\right]}_{1}, \end{array}$$ (24)

where [⋅]_1_ is the first entry of a vector (but it can be any other entry as well—they are all equal in the stationary case).

Having computed the stationary distribution of the queue length, we can compute the loss ratio of the system. The loss ratio is the fraction of customers rejected upon arrival, measured in a long time interval. It will be denoted by *L*. Its relation with the empty system probability is not difficult to derive. Namely, in a long time interval of length *T*, the service station is busy for approximately (1−*P*_0_)*T* time. As $\int_{0}^{\infty }xdF\left(x\right)$ is the average service time, the number of customers served in a long interval of length *T* is approximately $\left(1-P_{0}\right)T/\int_{0}^{\infty }xdF\left(x\right).$ On the other hand, there are approximately Λ*T* new customers arriving to the system in a long interval of length *T*. Therefore, the loss ratio must be:

$$\begin{array}{c} L=1-\frac{(1-P_{0})T}{\int_{0}^{\infty }xdF\left(x\right)}/\left(\Lambda \;T\right)=1-\frac{1-P_{0}}{\rho }. \end{array}$$ (25)

## 4 Auxiliary results

In this section, functions *H*_*n*,*k*,*i*,*j*_(*t*) and *q*_*n*_(*v*,*k*) for the BMAP process and the dropping mechanism will be computed. This is the last step necessary to use Theorem 1 in numerical calculations.

Denoting

$$\begin{array}{c} h_{n,k,i,j}\left(s\right)=\int_{0}^{\infty }e^{-st}H_{n,k,i,j}\left(t\right)dt, \end{array}$$

$$\begin{array}{c} h_{n,k}\left(s\right)={\left[h_{n,k,i,j}\left(s\right)\right]}_{i,j},\;\;\;\;1\leq i,j\leq m, \end{array}$$

we will prove first the following theorem.

**Theorem 2.** *It holds*

$$\begin{array}{c} h_{n,0}\left(s\right)=Y_{n}\left(s\right)Z\left(s\right),\;\;\;0\leq n\leq b, \end{array}$$ (26)

$$\begin{array}{c} h_{n,k}\left(s\right)=Y_{n}\left(s\right)\sum_{w=1}^{k}K_{n,w}\left(s\right)h_{n+w,k-w}\left(s\right),\;\;\;k\geq 1,\;0\leq n\leq b, \end{array}$$ (27)

*where h_n,k_*(*s*), *Y_n_*(*s*) *and* *Z*(*s*) *are the following m* × *m matrices*:

$$\begin{array}{c} Z(s)=diag(z(s)), \end{array}$$

$$\begin{array}{c} Y_{n}\left(s\right)={\left(I-K_{n,0}\left(s\right)\right)}^{-1}. \end{array}$$

proof. Consider first, that in time interval (0, *u*] one or more customers have been accepted to the waiting room. In this case, using the total probability formula with respect to the first event in BMAP, we can write for any *k* ≥ 1, 0 ≤ *n* ≤ *b*, 1 ≤ *i* ≤ *m*, 1 ≤ *j* ≤ *m* the following equation:

$$\begin{array}{cc} H_{n,k,i,j}\left(t\right)= & \sum_{a=1}^{m}\sum_{v=0}^{\infty }\sum_{w=0}^{k}\int_{0}^{t}q_{n}\left(v,w\right)p_{i}\left(v,a\right)\mu_{i}e^{-\mu_{i}u}H_{n+w,k-w,a,j}\left(t-u\right)du. \end{array}$$ (28)

The reasoning is somewhat similar to the one presented for Eq (7). The first event in BMAP happened after an exponentially distributed time with density *μ*_*i*_ *e*^−*μ*_*i*_*u*^. The first event could have been an arrival of a batch of size *v*, or a change of the modulating state into *a*, or both. What is important, the first event must have happened before *t*. If a batch of *v* customers arrived, *w* of these customers were accepted with probability *q*_*n*_(*w*, *k*). Therefore, at time *u* we had *n*+*w* customers in the system and the state of the modulating chain was *a*.

Now, consider that no customers have been accepted in (0, *u*]. In this case, the first event in (0, *u*] could have been either a change of the modulating state (without arrival), or an arrival of a batch of customers, of which all customers were rejected. Moreover, it could have happened that the first event was after *t*. Therefore, we have:

$$\begin{array}{ll} H_{n,0,i,j}\left(t\right)= & \sum_{a=1}^{m}\sum_{v=0}^{\infty }\overset{}{\underset{}{\int_{0}^{t}q_{n}\left(v,0\right)p_{i}\left(v,a\right)\mu_{i}e^{-\mu_{i}u}H_{n,0,a,j}\left(t-u\right)du}}\\ & +\delta_{ij}e^{-\mu_{i}t}, \end{array}$$ (29)

for 0 ≤ *n* ≤ *b*, 1 ≤ *i* ≤ *m*, 1 ≤ *j* ≤ *m*.

Application of the Laplace transform to Eqs (28) and (29) yields:

$$\begin{array}{c} h_{n,k,i,j}\left(s\right)=\sum_{a=1}^{m}\sum_{v=0}^{\infty }\sum_{w=0}^{k}\frac{q_{n}\left(v,w\right)p_{i}\left(v,a\right)\mu_{i}}{s+\mu_{i}}h_{n+w,k-w,a,j}\left(s\right),\;\;\;k\geq 1, \end{array}$$ (30)

and

$$\begin{array}{c} h_{n,0,i,j}\left(s\right)=\sum_{a=1}^{m}\sum_{v=0}^{\infty }\frac{q_{n}\left(v,0\right)p_{i}\left(v,a\right)\mu_{i}}{s+\mu_{i}}h_{n,0,a,j}\left(s\right)+\frac{\delta_{ij}}{s+\mu_{i}}, \end{array}$$ (31)

respectively. Applying the matrix notation to Eq (30) we get:

$$\begin{array}{c} h_{n,k}\left(s\right)=\sum_{w=0}^{k}K_{n,w}\left(s\right)h_{n+w,k-w}\left(s\right),\;\;\;k\geq 1, \end{array}$$ (32)

while Eq (31) yields:

$$\begin{array}{c} h_{n,0}\left(s\right)=K_{n,0}\left(s\right)h_{n,0}\left(s\right)+Z\left(s\right). \end{array}$$ (33)

This finishes the proof, as Eq (32) is equivalent to Eq (27), while Eq (33) is equivalent to Eq (26).

As for the numerical calculations of *H*_*n*,*k*,*i*,*j*_(*u*) values from *h*_*n*,*k*,*i*,*j*_(*s*), one can use the aforementioned methods of transform inversion [52–56]. We use the Spinelli method of [52].

Calculating *q*_*n*_(*v*,*k*) is straightforward. We obviously have:

$$\begin{array}{c} q_{n}\left(v,k\right)=0,\;\;\;k>\min\left\{b-n,v\right\}, \end{array}$$ (34)

and

$$\begin{array}{c} q_{n}\left(0,k\right)=0,\;\;\;k>0, \end{array}$$ (35)

and

$$\begin{array}{c} q_{n}\left(v,0\right)={\left(d(n)\right)}^{v},\;\;\;v>0, \end{array}$$ (36)

and

$$\begin{array}{c} q_{n}\left(0,0\right)=1. \end{array}$$ (37)

Finally, we obtain:

$$\begin{array}{c} q_{n}\left(v,k\right)=d\left(n\right)q_{n}\left(v-1,k\right)+\left(1-d\left(n\right)\right)q_{n+1}\left(v-1,k-1\right),\;\;\;v>0,k>0. \end{array}$$ (38)

## 5 Examples

For numerical purposes, we use the following BMAP parameterization, [50]:

$$\begin{array}{ccc} D_{0} & = & \left[\begin{array}{ccc} -0.0499514 & \;\;0.00399715 & \;\;0.00128940\\ 0.00528656 & \;\;-0.0774334 & \;\;0.00528656\\ 0.00141834 & \;\;0.00141834 & \;\;-0.274511 \end{array}\right], \end{array}$$

$$\begin{array}{ccc} D_{1} & = & \left[\begin{array}{ccc} 0.0181806 & \;\;0.00141834 & \;\;0.00270775\\ 0.00141834 & \;\;0.00399715 & \;\;0.00270775\\ 0.00270775 & \;\;0.00399715 & \;\;0.00657596 \end{array}\right], \end{array}$$

$$\begin{array}{ccc} D_{4} & = & \left[\begin{array}{ccc} 0.00141834 & \;\;0.00270775 & \;\;0.00141834\\ 0.00141834 & \;\;0.0413899 & \;\;0.00141834\\ 0.00528656 & \;\;0.00141834 & \;\;0.00270775 \end{array}\right], \end{array}$$

$$\begin{array}{ccc} D_{10} & = & \left[\begin{array}{ccc} 0.00483682 & \;\;0.00714007 & \;\;0.00483682\\ 0.00253357 & \;\;0.00944332 & \;\;0.00253357\\ 0.00714007 & \;\;0 & \;\;0.241841 \end{array}\right]. \end{array}$$

As can be easily computed, the average rate of this BMAP is 1, while the average size of an arriving batch is 8. Moreover, the process is strongly autocorrelated (see [50] for the graph of its autocorrelation function). It is assumed that the capacity of the waiting room is 200, i.e. *b* = 200. If not stated otherwise, the service time is 1.1, which gives the load of the system equal to 110%—the system is overloaded.

To demonstrate capabilities of dropping functions, we will present several functions providing arbitrary values of the average queue length, as well as arbitrary values of the loss ratio. Then a function that keeps two different values of the average queue length, depending on the load of the system, will be presented.

Firstly, how can we obtain an arbitrary value of the average queue length? To show this, let us start with the class of simple linear dropping functions in the form:

$$\begin{array}{c} d_{a}\left(n\right)=\left\{\begin{array}{ccc} 0, & \;\text{if}\; & n<0,\\ a\;n, & \;\text{if}\; & 0\leq n<200\;\;\text{and}\;\;a\;n<1,\\ 1, & \;\text{if}\; & n\geq 200\;\;\text{or}\;\;a\;n\geq 1, \end{array}\right. \end{array}$$

where *a* is a parameter. Let us assume also, that the required average queue length is *x*. If $E_{d_{a}}\left(\Phi \right)$ denotes the average queue length for dropping function *d*_*a*_ (which can be computed using Theorem 1, then we have to solve numerically the following equation:

$$\begin{array}{c} E_{d_{a}}\left(\Phi \right)=x, \end{array}$$

with respect to *a*. This equation can be solved, for instance, using the bisection method.

**Fig 2:**
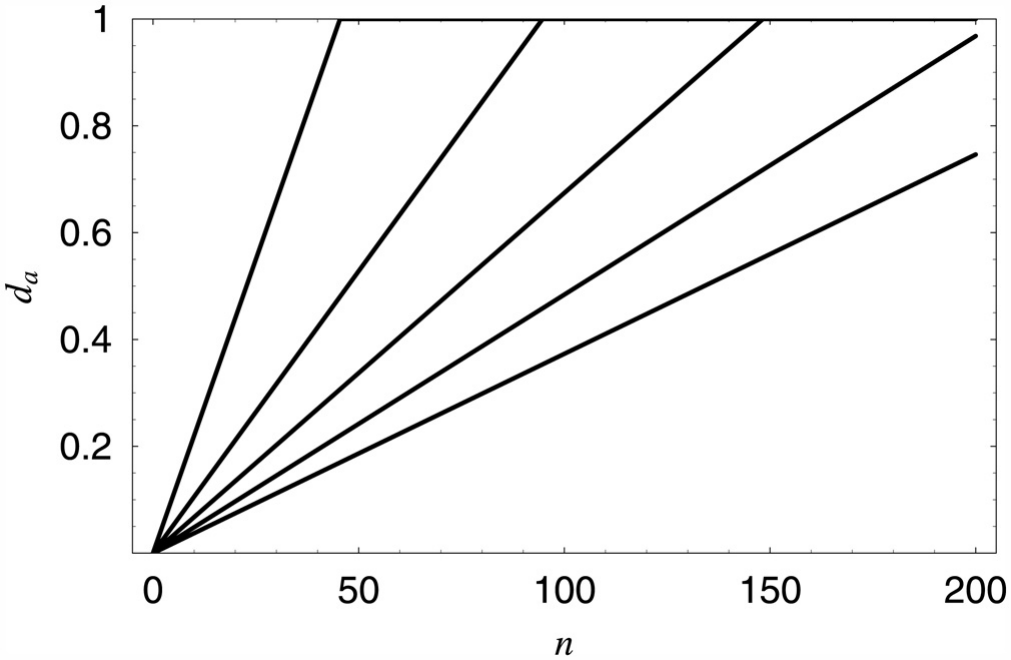
Five linear dropping functions providing the average queue length of 50, 40, 30, 20 and 10 customers, respectively (counting from the bottom).

**Fig 3:**
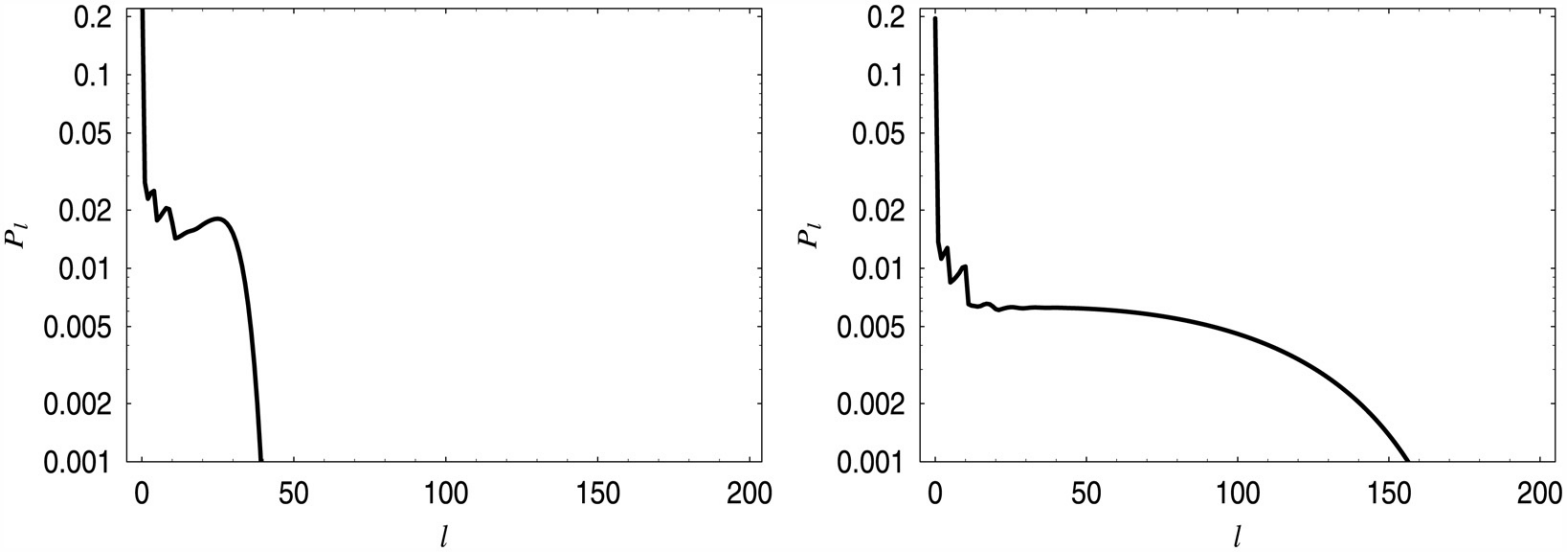
Queue length distributions for dropping function *d*_*a*_. On the left, for *a* = 0.02199, on the right, for *a* = 0.0037315.

In Table 1, five computed values of *a* are presented. They were obtained assuming that the required average queue length is *x* = 10.0, 20.0, 30.0, 40.0 and 50.0 customers, respectively. In Fig 2, the five linear dropping functions with the values of *a* from Table 1 are depicted. Finally, in Fig 3 the distributions of the queue length for the first and the last dropping function from Table 1 are depicted. The spikes, which can be observed in low queue ranges, are typical when a batch arrivals are involved. They are connected with possible batch sizes—herein 1, 4, and 10—and their combinations.

**Table 1:** Performance characteristics of systems with dropping function *d*_*a*_ for five values of *a*.

*a*	average queue length	std. dev. queue length	loss ratio
0.0219900	**10.0**	11.4	45.0%
0.0105600	**20.0**	21.0	37.3%
0.0067440	**30.0**	29.6	32.6%
0.0048400	**40.0**	37.9	29.3%
0.0037315	**50.0**	45.6	26.9%

Arbitrary values of the loss ratio can be achieved in the same way as the queue lengths. To show this, let us consider the following class of quadratic dropping functions:

$$\begin{array}{c} d_{b}\left(n\right)=\left\{\begin{array}{ccc} 0, & \;\text{if}\; & n<0,\\ bn^{2}, & \;\text{if}\; & 0\leq n<200\;\;\text{and}\;\;bn^{2}<1,\\ 1, & \;\text{if}\; & n\geq 200\;\;\text{or}\;\;b\;n^{2}\geq 1, \end{array}\right. \end{array}$$

where *b* is a parameter.

Let *L*_*d*_*b*__ denote the loss ratio for dropping function *d*_*b*_ (which can be computed using Eq (25) and Theorem 1) and *y* be the required loss ratio. Thus we have to solve numerically the following equation:

$$\begin{array}{c} L_{d_{b}}=y, \end{array}$$

with respect to *b*. This again can be done using bisections.

For instance, the obtained five values of *b* which provide the loss ratio of *y* = 20.0%, 25.0%, 30.0%, 35.0% and 40.0%, are presented in in Table 2, respectively. They are accompanied with basic performance characteristics of systems with dropping functions *d*_*b*_.

**Table 2:** Performance characteristics of systems with dropping function *d*_*b*_ for five values of *b*.

*b*	average queue length	std. dev. queue length	loss ratio
0.00000935	88.3	64.8	**20.0%**
0.00003180	54.4	45.2	**25.0%**
0.00007900	34.6	31.2	**30.0%**
0.00018170	22.6	22.0	**35.0%**
0.00042000	14.6	15.1	**40.0%**

**Fig 4:**
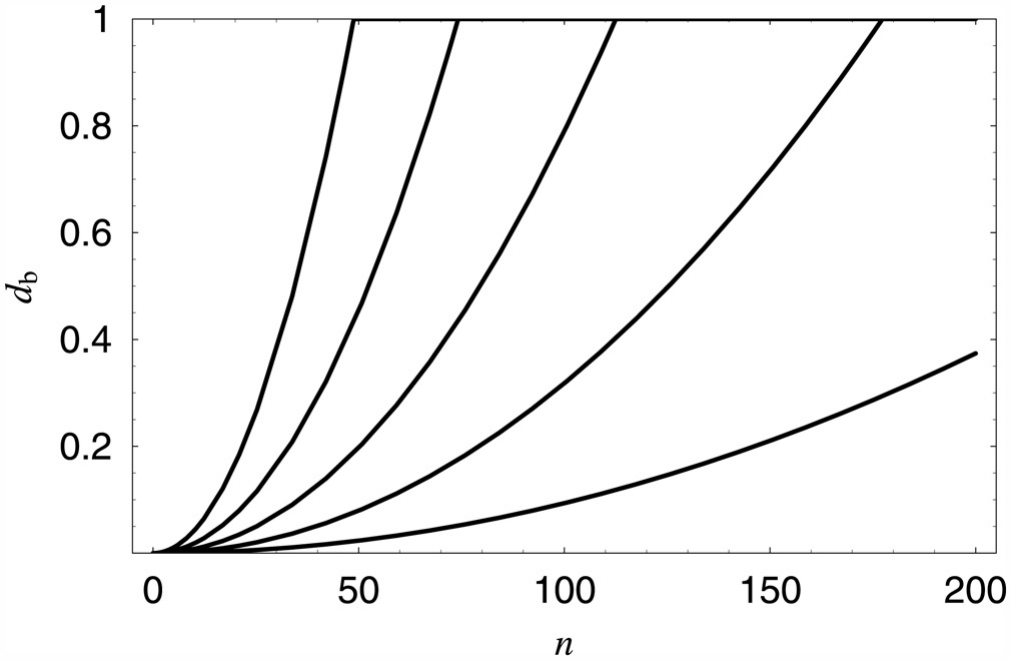
Five dropping functions providing the loss ratio of 20%, 25%, 30%, 35% and 40%, respectively (counting from the bottom).

**Fig 5:**
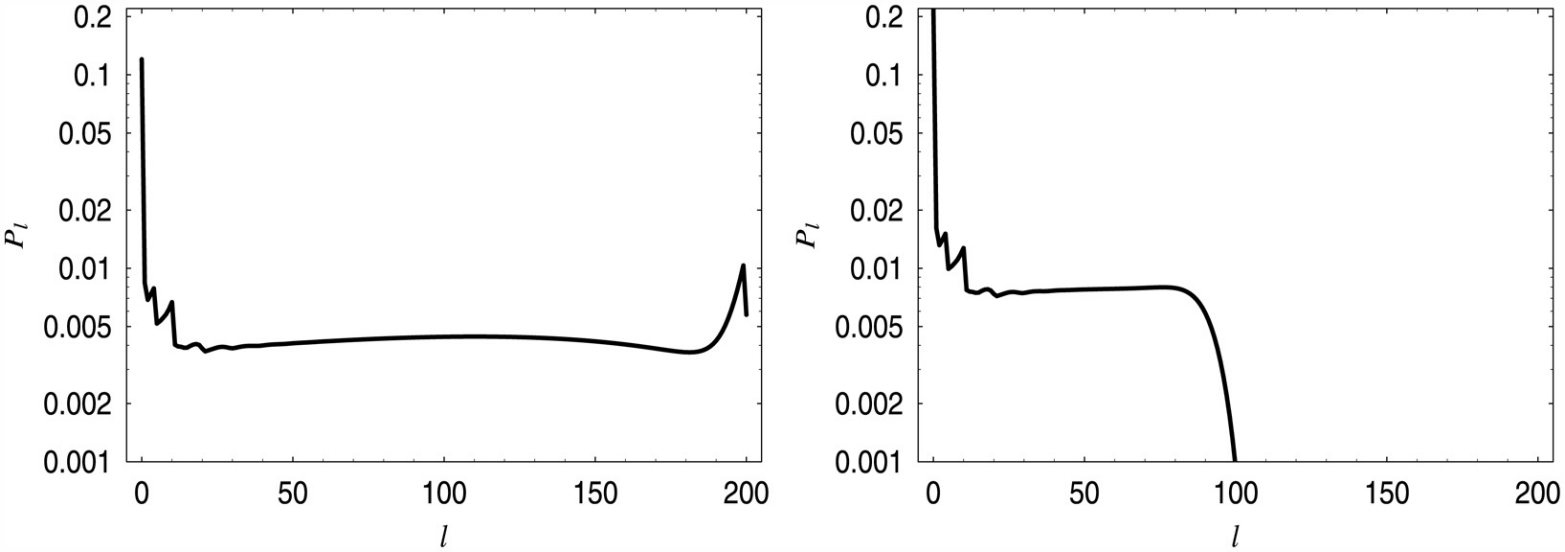
Queue length distributions for dropping function *d*_*b*_. On the left, for *b* = 0.00000935, on the right, for *b* = 0.00007900.

In addition, in Fig 4 all quadratic functions with the values of *b* from Table 2 are depicted, while in Fig 5 the distributions of the queue length for the first and the third dropping function from Table 2 are depicted.

Two questions arise after the first two examples.

Firstly, what are the minimum and maximum values of the average queue length and the loss ratio that can be obtained using the dropping function? To answer this, we have to compute the performance characteristics of the system without the dropping function, or, in other words, with the trivial dropping function in the form:

$$\begin{array}{c} d\left(n\right)=\left\{\begin{array}{ccc} 0, & \;\text{for}\; & n<200,\\ 1, & \;\text{for}\; & n\geq 200. \end{array}\right. \end{array}$$

The resulting average queue length obtained from Theorem 1 is 103.1, while the loss ratio is 18.1%. Therefore, using dropping functions we can obtain any average queue length in interval [0, 103.1] and any loss ratio in interval [18.1%, 100%].

It is a simple matter to check, that in the second example we could have also used linear dropping functions to obtain loss ratios of 20%, 25%, 30%, etc. (This can be verified using bisections for functions *d*_*a*_).

This observation leads to the second question. If the simple, linear dropping functions seem to have full control capabilities regarding the average queue length and the loss ratio, then what is the use of more complicated shapes of dropping functions?

To answer that, we will show first that there are many different dropping functions providing the same average queue length, but different other performance characteristics (e.g. the loss ratio).

**Fig 6:**
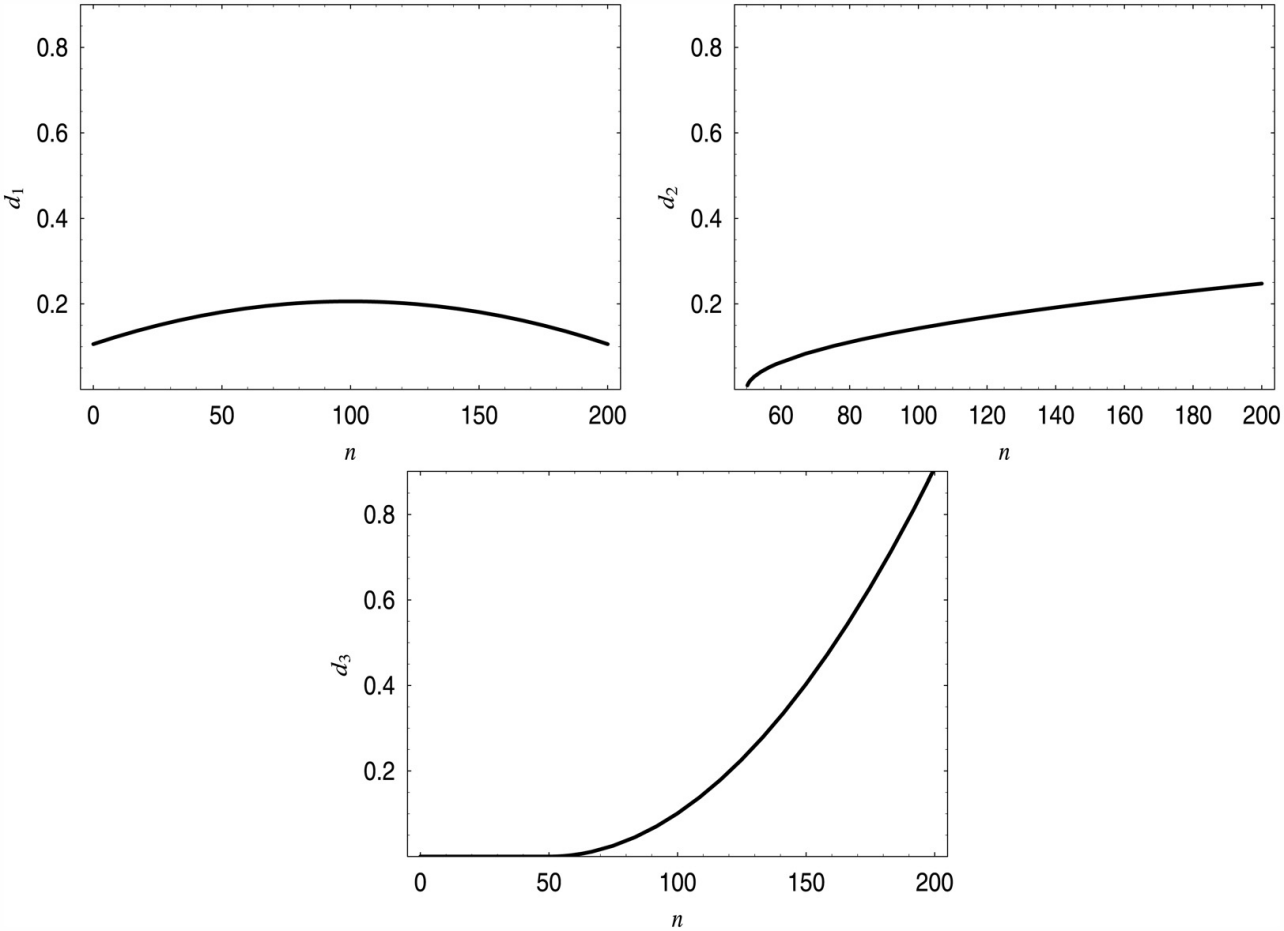
Three different dropping functions providing the average queue length of 75 customers.

For instance, the following three dropping functions were found, each of them providing the average queue length of 75:

$$\begin{array}{c} d_{1}\left(n\right)=\left\{\begin{array}{ccc} 0, & \;\text{for}\; & n<0,\\ -0.00001{\left(n-100\right)}^{2}+0.20597, & \;\text{for}\; & 0\leq n<200,\\ 1, & \;\text{for}\; & n\geq 200, \end{array}\right. \end{array}$$

$$\begin{array}{c} d_{2}\left(n\right)=\left\{\begin{array}{ccc} 0, & \;\text{for}\; & n<0,\\ 0.02021018\sqrt{n}, & \;\text{for}\; & 0\leq n<200,\\ 1, & \;\text{for}\; & n\geq 200, \end{array}\right. \end{array}$$

$$\begin{array}{c} d_{3}\left(n\right)=\left\{\begin{array}{ccc} 0, & \;\text{for}\; & n\leq 50,\\ 0.00004035{\left(n-50\right)}^{2}, & \;\text{for}\; & 50<n<200,\\ 1, & \;\text{for}\; & n\geq 200. \end{array}\right. \end{array}$$

(In the case of dropping functions with two parameters we can set one parameter manually and find the other using bisesctions). The shapes of these dropping functions are presented in Fig 6. As we can see, they are quite different—one function is non-monotonic, two functions are monotonic, one function is convex, two are concave.

In Table 3, the performance characteristics of the systems with dropping functions *d*_1_–*d*_3_ are presented. As we can see, the average queue length is common, but other characteristics are different.

**Table 3:** Performance characteristics of systems with dropping functions *d*_1_–*d*_3_.

dropping function	average queue length	std. dev. queue length	loss ratio
*d*_1_	**75.0**	65.6	23.9%
*d*_2_	**75.0**	63.5	22.8%
*d*_3_	**75.0**	56.5	21.2%

**Fig 7:**
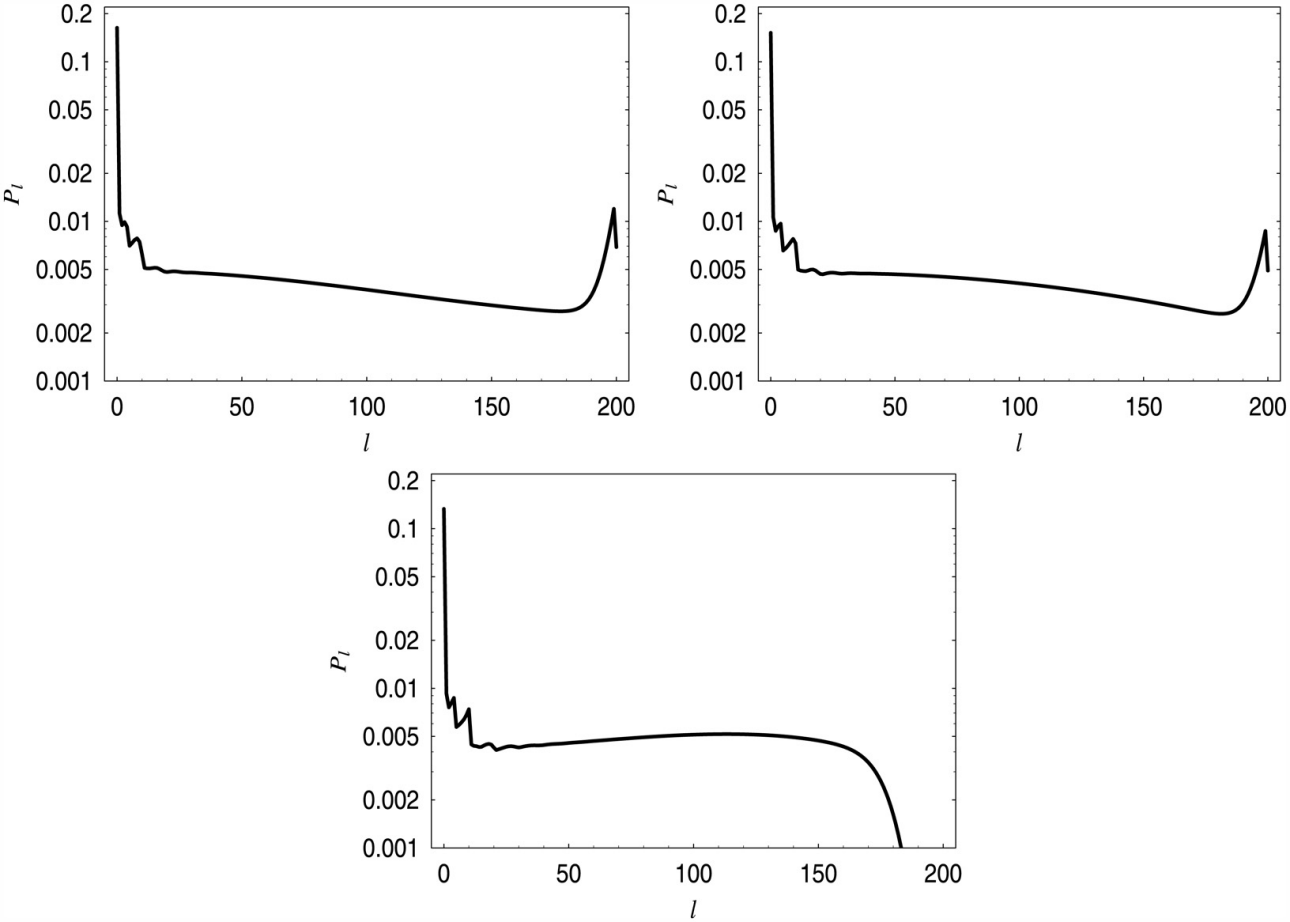
Queue length distributions for dropping functions *d*_1_–*d*_3_. Distribution for *d*_1_ in the upper-left corner, for *d*_2_ in the upper-right corner, for *d*_3_ in the bottom.

In Fig 7, distributions of the queue length in systems with dropping functions *d*_1_–*d*_3_ are presented.

Similarly, there are many different dropping functions providing the same value of the loss ratio, but different other performance characteristics (e.g. the queue length). For instance, in addition to the first dropping function in Table 2, the following three different dropping functions provide the customer loss ratio of 20%:

$$\begin{array}{c} d_{4}\left(n\right)=\left\{\begin{array}{ccc} 0, & \;\text{for}\; & n<0,\\ 0.22606729e^{-{\left(n-120\right)}^{2}/1800}, & \;\text{for}\; & 0\leq n<200,\\ 1, & \;\text{for}\; & n\geq 200, \end{array}\right. \end{array}$$

$$\begin{array}{c} d_{5}\left(n\right)=\left\{\begin{array}{ccc} 0, & \;\text{for}\; & n<0,\\ \left(n/12000\right)\left(n/12500\right)\left|sin\left(n\right)\right|+0.063 & \;\text{for}\; & 0\leq n<200,\\ 1, & \;\text{for}\; & n\geq 200, \end{array}\right. \end{array}$$

$$\begin{array}{c} d_{6}\left(n\right)=\left\{\begin{array}{ccc} 0, & \;\text{for}\; & n<0,\\ 0.0005n & \;\text{for}\; & 0\leq n<120,\\ 0.0063n-0.696 & \;\text{for}\; & 120\leq n<200,\\ 1, & \;\text{for}\; & n\geq 200. \end{array}\right. \end{array}$$

**Fig 8:**
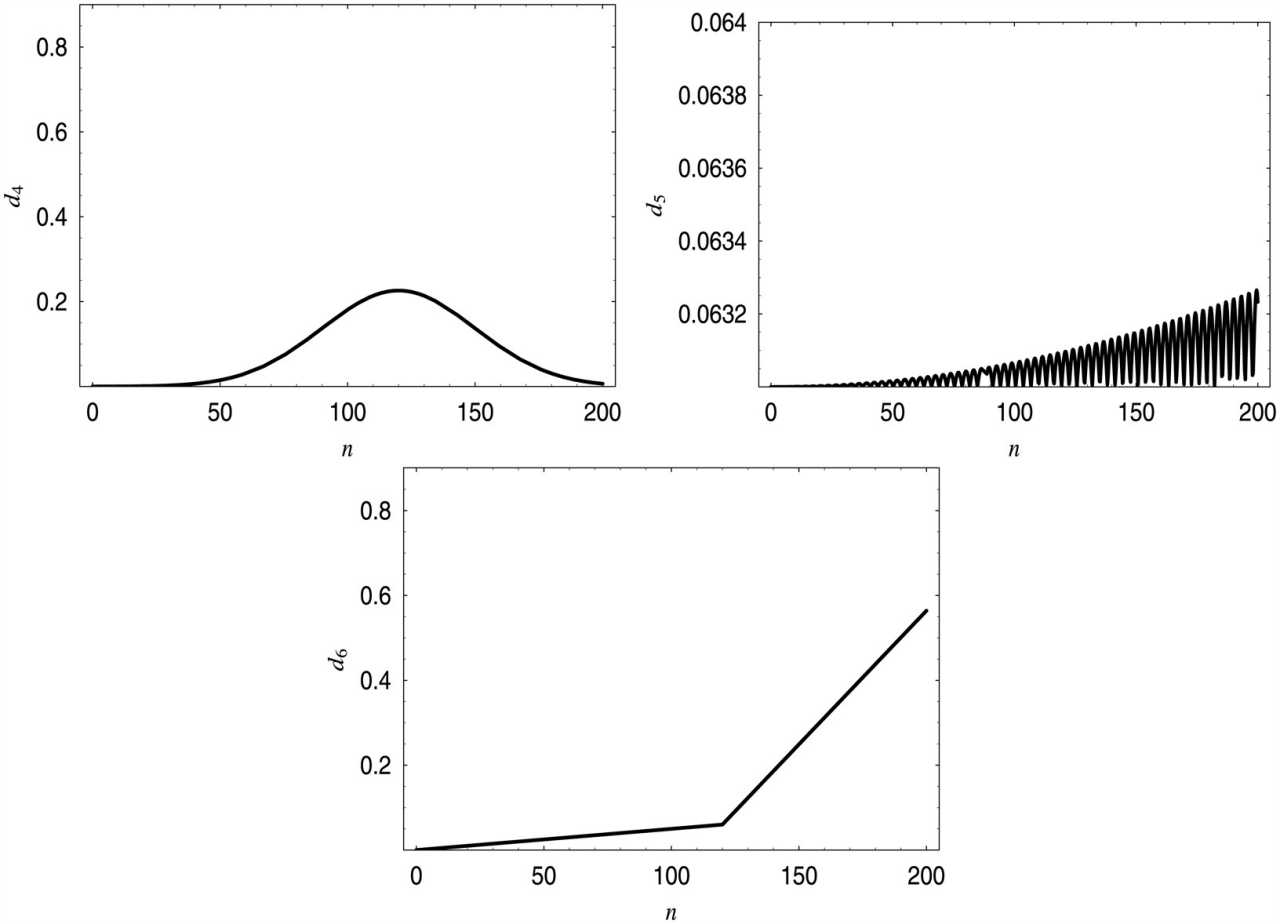
Three different dropping functions providing the loss ratio of 20% of customers.

**Fig 9:**
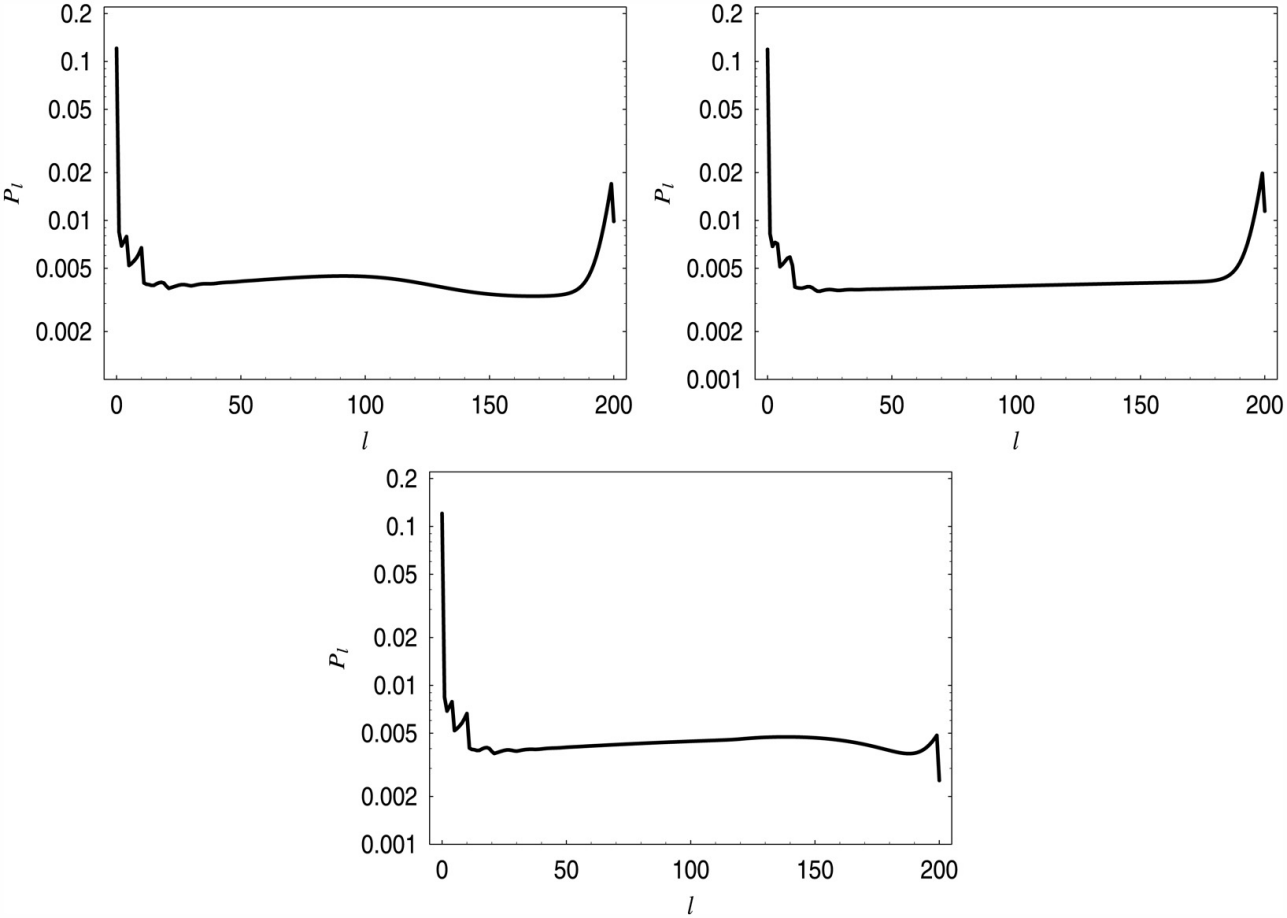
Queue length distributions for dropping functions *d*_4_–*d*_6_. Distribution for *d*_4_ in the upper-left corner, for *d*_5_ in the upper-right corner, for *d*_6_ in the bottom.

The shapes of these functions are depicted in Fig 8, while in Table 4 the performance characteristics of the systems exploiting them are shown. In Fig 9, distributions of the queue length in systems with dropping functions *d*_4_–*d*_6_ are depicted.

**Table 4:** Performance characteristics of systems with dropping functions *d*_4_–*d*_6_.

dropping function	average queue length	std. dev. queue length	loss ratio
*d*_4_	88.1	66.6	**20.0%**
*d*_5_	94.2	68.7	**20.0%**
*d*_6_	86.8	63.3	**20.0%**

As we can see, the queue length and its deviation can vary, even if the loss ratio is common in all three systems.

We can conclude that using different shapes of dropping function opens the possibility to control more than one performance characteristics at the same time. (However, every next characteristic in a smaller interval).

In the final example, the dropping function was designed is such a way, that it provides the average queue length of 30 customers when the load of the system is 90% (underloaded system), and 60 customers—when the load is 110% (overloaded system).

**Fig 10:**
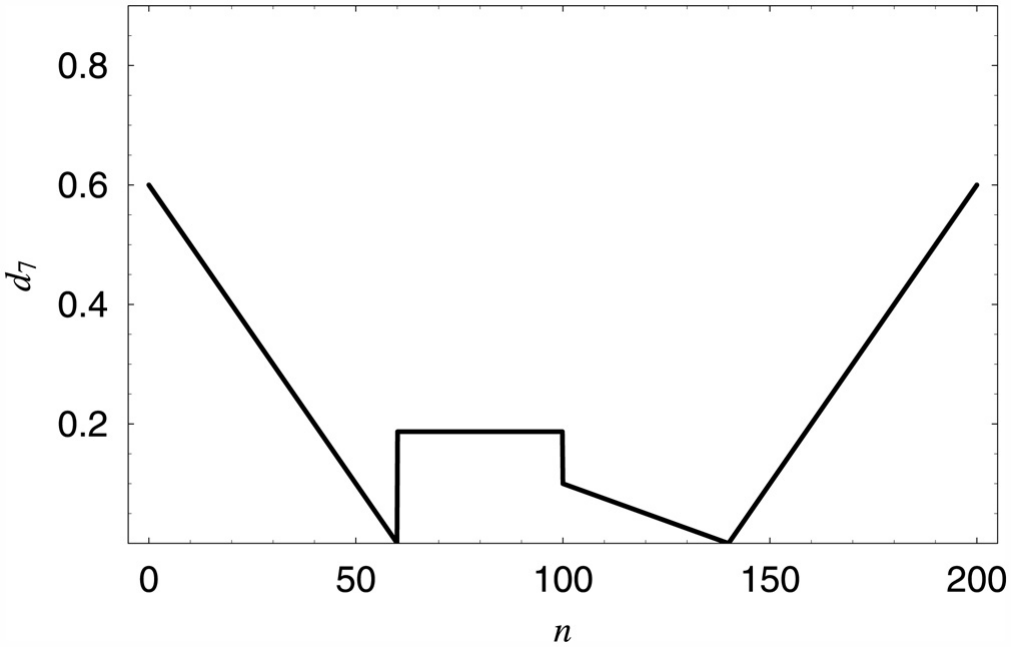
Dropping function providing the average queue length of 30 customers for *ρ* = 90%, and 60 customers for *ρ* = 110%.

To find the parameters of the dropping function in this case, the system of two equations had to be solved numerically. The obtained function has the following form:

$$\begin{array}{c} d_{7}\left(n\right)=\left\{\begin{array}{ccc} 0, & \;\text{for}\; & n<0,\\ -0.01+0.6n & \;\text{for}\; & 0\leq n<60,\\ 0.187 & \;\text{for}\; & 60\leq n<100,\\ -0.0025(n-140) & \;\text{for}\; & 100\leq n<140,\\ 0.01(n-140) & \;\text{for}\; & 140\leq n<200,\\ 1, & \;\text{for}\; & n\geq 200, \end{array}\right. \end{array}$$

and is depicted in Fig 10.

**Fig 11:**
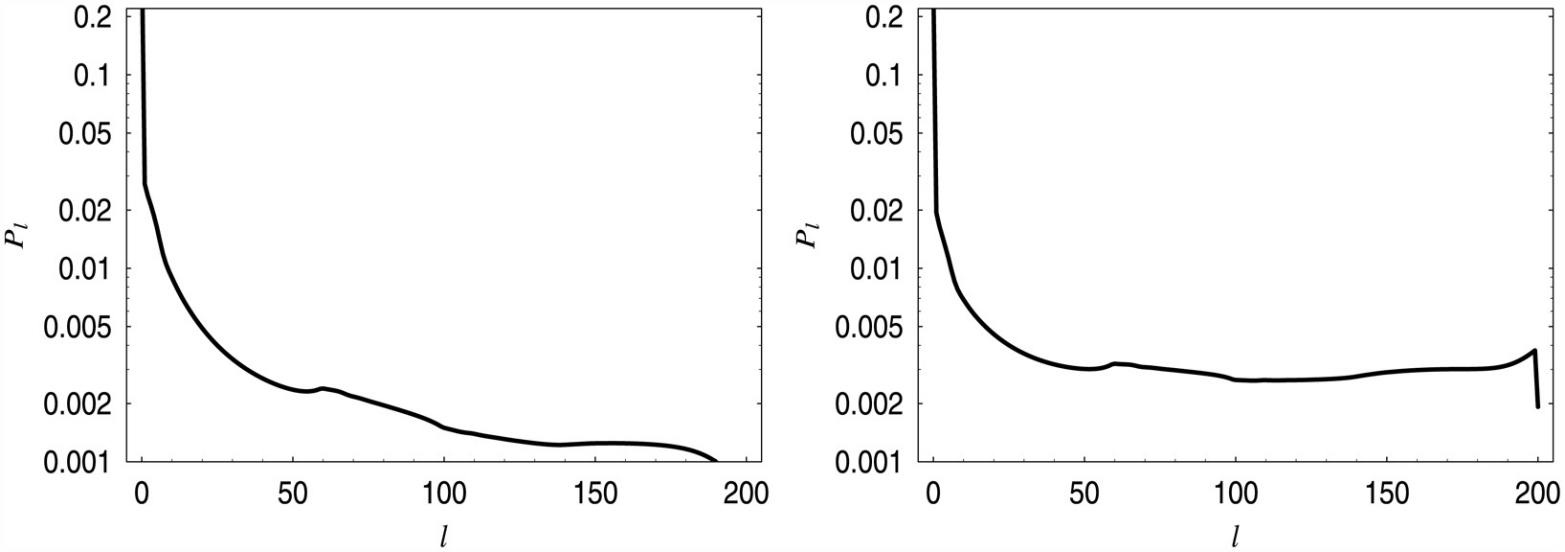
Queue length distributions for dropping function *d*_7_. On the left, for *ρ* = 90%, on the right, for *ρ* = 110%.

The performance characteristics for *d*_7_ and different loads are given in Table 5, while the distributions of the queue length are shown in Fig 11.

**Table 5:** Performance characteristics of systems with dropping function *d*_7_ and two different loads.

dropping function	average queue length	std. dev. queue length	loss ratio
*d*_7_, *ρ* = 90%	**30.0**	50.1	39.8%
*d*_7_, *ρ* = 110%	**60.0**	65.9	35.3%

This example demonstrates the possibility of designing the dropping function in such a way, that for different loads it keeps performance characteristics at some arbitrary levels.

## 6 Conclusions

We presented an analysis of the queueing system with the dropping function of arbitrary type, arbitrary distribution of the service time, and a very general customer arrival process, which allows for modeling of autocorrelation, batch arrivals and arbitrary shape of the interarrival time distribution. We obtained the distribution of the queue length both in transient and stationary regime, computed the loss ratio and presented several numerical examples.

As for the future work and open questions, probably the most interesting is the question on the stability condition for the systems with BMAP arrivals and infinite waiting room. Naturally, if the dropping function is applied in a system with infinite waiting room, the stability condition is not *ρ* < 1 any more. For instance, if *ρ* = 2 and the dropping function is *d*(*n*) = 0.51 for every *n*, then the system is obviously stable. But would it be stable for a more sophisticated dropping function, say $d\left(n\right)=0.51-\frac{1}{n}$, *n* ≥ 2? Does the stability depend only on *ρ* and the dropping function or on the BMAP structure as well?
